# Recognition and Misclassification Patterns of Basic Emotional Facial Expressions: An Eye-Tracking Study in Young Healthy Adults

**DOI:** 10.3390/jemr18050053

**Published:** 2025-10-11

**Authors:** Neşe Alkan

**Affiliations:** Department of Psychology, Atılım University, Ankara 06830, Turkey; nese.alkan@atilim.edu.tr

**Keywords:** facial emotion recognition, eye-tracking, gaze allocation, misclassification, heatmaps, areas of interest, gender differences

## Abstract

Accurate recognition of basic facial emotions is well documented, yet the mechanisms of misclassification and their relation to gaze allocation remain under-reported. The present study utilized a within-subjects eye-tracking design to examine both accurate and inaccurate recognition of five basic emotions (anger, disgust, fear, happiness, and sadness) in healthy young adults. Fifty participants (twenty-four women) completed a forced-choice categorization task with 10 stimuli (female/male poser × emotion). A remote eye tracker (60 Hz) recorded fixations mapped to eyes, nose, and mouth areas of interest (AOIs). The analyses combined accuracy and decision-time statistics with heatmap comparisons of misclassified versus accurate trials within the same image. Overall accuracy was 87.8% (439/500). Misclassification patterns depended on the target emotion, but not on participant gender. Fear male was most often misclassified (typically as disgust), and sadness female was frequently labeled as fear or disgust; disgust was the most incorrectly attributed response. For accurate trials, decision time showed main effects of emotion (*p* < 0.001) and participant gender (*p* = 0.033): happiness was categorized fastest and anger slowest, and women responded faster overall, with particularly fast response times for sadness. The AOI results revealed strong main effects and an AOI × emotion interaction (*p* < 0.001): eyes received the most fixations, but fear drew relatively more mouth sampling and sadness more nose sampling. Crucially, heatmaps showed an upper-face bias (eye AOI) in inaccurate trials, whereas accurate trials retained eye sampling and added nose and mouth AOI coverage, which aligned with diagnostic cues. These findings indicate that the scanpath strategy, in addition to information availability, underpins success and failure in basic-emotion recognition, with implications for theory, targeted training, and affective technologies.

## 1. Introduction

Facial expression of emotions constitutes one of the most immediate and universal channels of communication [1,2,3]. Humans mainly rely on visual cues to infer the psychological state of others as a function of their facial expressions [4]. Morphological changes in the face, such as widening the eyes, pulling the lip corners upward, frowning, and nose wrinkling serve, as diagnostic indicators [5]. Accurate recognition of basic emotions is central to effective interpersonal communication and adaptive behavior [6,7]. Emotions that are expressed and recognized universally are called basic emotions. Ekman [8] argued that there are six basic emotions, anger, disgust, fear, happiness, sadness, and surprise, each corresponding to distinctive facial expressions. These emotions are also called universal emotions because they are not only expressed with consistent and distinctive features but are also recognized universally.

Research across psychology, affective computing, and psychotherapy has consistently emphasized accurate recognition of basic emotions [9]. Since it is an important aspect of social cognition, which refers to the ability to perceive other people’s intentions and temperament and to subsequently guide and regulate behaviors and social activities, prior research has extensively documented the accuracy of facial emotion recognition across various populations [10] and age groups, including children [11]. Growing evidence suggests that some individuals systematically misclassify certain expressions. For example, disgust is frequently mistaken for anger, suggesting that errors in recognition are not random but follow predictable patterns. Such misperceptions have significant real-world consequences. Misperceiving another person’s emotional state may hinder empathy, lead to interpersonal conflict, or impair emotional regulation in dyadic interactions, including romantic relationships and even psychotherapy [12,13]. In addition to its importance at the interpersonal level, accurate or inaccurate emotion recognition may also play a role at the societal level, such as in shaping stereotypical beliefs about groups [14].

Although much is known about accuracy in facial emotion recognition, less is understood about the mechanisms that give rise to errors. The extant literature on inaccurate emotion recognition relied heavily on clinical populations. Due to its debilitating effect on interpersonal and social functioning [15], facial emotion recognition impairment has been extensively studied in various psychiatric conditions, such as schizophrenia [4] depression [16], mild cognitive impairment [17], Parkinson’s disease [18], and autism spectrum disorder. Despite the number and variety of studies in clinical populations, the reasons and clear patterns of inaccurate emotion recognition remain to be fully understood, partly due to the complex neural basis of facial recognition deficits [19].

Patterns and processes of inaccurate emotion recognition in healthy individuals, on the other hand, remain an under-represented research area. Earlier research on recognition of facial expression indicated that distinct patterns of eye fixation distributions over certain facial features are associated with certain emotions. A widely supported pattern indicates that the recognition of anger, fear, and sadness depends primarily on cues from the upper face (e.g., brows, eyes), while happiness and disgust are identified mainly through features in the lower face (e.g., mouth, nose) [20]. Recent work, on the other hand, has emphasized that successful recognition of facial expressions can emerge from diverse fixation distributions rather than normative gaze strategy [21]. In parallel, gaze allocation has been shown to reflect idiosyncratic patterns that generalize across both static and dynamic facial stimuli, suggesting that recognition outcomes are partly shaped by individual visual strategies [22]. Growing evidence suggests that healthy individuals also frequently misclassify certain emotions, particularly disgust, fear, and anger [23,24,25,26,27,28,29]. The spatial distribution of eye movements has also been linked to systematic misclassifications of emotional expressions, highlighting the role of attentional biases. For example, Paparelli et al. [22] demonstrated a key distinction in the diagnostic value of facial regions for anger: higher fixation on the eyes correlated with higher misclassification rates, whereas greater attention to the mouth region facilitated more accurate recognition. It was shown that failed recognition attempts are associated with a generalized increase in fixation duration, and not limited to diagnostic features of the facially expressed emotions [23]. This suggests that observers adopt a less selective attentional strategy when uncertain, scrutinizing both relevant and irrelevant facial areas. In another experimental study, researchers reported unexpected results, such as the absence of an association between the outer brow raiser and suddenness, and an unexpected association between the lower lip depressor and fear and goal obstruction [29]. A meta-analytic investigation, on the other hand, indicated attentional bias for positive emotional stimuli, reporting larger biases for positive stimuli in paradigms measuring early, rather than late, attentional processing [30]. Although this study suggested that attentional bias for positive stimuli occurs rapidly and involuntarily, attentional processes underlying the recognition of basic emotional facial expressions—including negative ones—remain to be further explored.

Regarding gender differences in emotion recognition, studies have produced mixed results. Some studies have found that females have a generally higher rate of identification accuracy than males [31] and are particularly better at the explicit identification of fear [28]. Other studies, however, have indicated no gender difference. For example, Lambrecht et al. [32] reported no difference in expressed-emotion recognition accuracy. The researchers noted that the only gender difference observed was in the recognition of emotional prosody, which was mediated by participants’ hearing loss. Similarly, several other experiments reported that their experimental results did not show a significant effect of gender on emotion recognition variables (e.g., [23,33]). Given this lack of consensus, examining whether gender differences extend not only to recognition accuracy but also to misclassification patterns and underlying attentional strategies provides an important avenue for clarifying the role of gender in emotion perception.

Based on the above literature, the purpose of the current study is to examine both inaccurate and accurate recognition of facial emotion expressions in young healthy adults using an experimental approach. The study is grounded in the information-processing perspective of expression recognition [34], which posits that the processing of physical facial features and their configurations leads to the assignment of category labels such as fear, anger, or happiness [35]. The experimental task used was a categorization task, operationalized as the assignment of a facial stimulus to an emotion or expression category [36]. In this task, participants match their responses with predefined categories. In the present study, as part of the classification task, participants viewed photographs of facial expressions from a widely used set of emotion expressions depicting five basic emotions—anger, disgust, fear, happiness, and sadness—displayed by one male and one female poser.

Although surprise has been widely considered a basic emotion in early theoretical frameworks [2], its status remains a matter of ongoing debate [37]. Several scholars argue that surprise may not be considered basic because of certain characteristics, such as its link to expectation violation, brief duration [38], valence ambiguity [39], lack of self-containment, and knowledge-based nature. Following this rationale, the present study focuses on five widely accepted basic emotions (anger, disgust, fear, happiness, and sadness) while excluding surprise, without dismissing its theoretical relevance. Furthermore, the study explores whether participant gender modulates attention allocation to different facial regions (e.g., eyes, nose, and mouth) during the emotion classification task. Finally, given that understanding where and how visual attention is allocated is central to vision science [40], the present study employed the eye-tracking methodology, a reliable tool in emotion research [41], to provide a multimodal perspective on emotion recognition.

Overall, this study addresses a notable gap in the literature. While prior research has focused heavily on clinical populations or relied primarily on overall accuracy scores, the systematic examination of both accurate and inaccurate classifications in healthy young adults remains limited. Furthermore, while recent work has established that successful recognition tolerates diverse fixation patterns [21,22] and that gaze distributions predict false recognition [23], few studies have directly combined behavioral accuracy measures with fine-grained eye-tracking analyses to compare attentional allocation during correct trials versus specific, common misclassifications. By adopting this multimodal approach, the present research contributes to a more nuanced understanding of how emotion recognition unfolds, where misclassifications emerge, and whether gender modulates these processes. Specifically, it investigates whether the known idiosyncrasy in gaze patterns for correct recognition contrasts with more systematic, error-driven patterns during misclassification. Clarifying these mechanisms is not only theoretically important for models of emotion processing and social cognition, but also has practical relevance for clinical interventions and human–computer interaction contexts where accurate emotion recognition is critical.

### Research Questions

Based on this rationale, the present study investigates how gaze allocation to facial features relates to both accurate and inaccurate recognition of basic emotions in healthy young adults. By integrating behavioral accuracy measures with eye-tracking data, the study addresses three key research questions: (1) Which basic emotions are most frequently misclassified, and what systematic misclassification patterns emerge? (2) Does participant gender influence recognition accuracy and decision time, and does it interact with the gender of the stimulus face? (3) Do different emotions elicit distinct gaze allocation patterns across the eyes, mouth, and nose, and are these patterns moderated by participant gender? By answering these questions, this study aims to advance two theoretical perspectives. First, by providing a more complete account of both accurate and inaccurate recognition of basic emotions, it extends the information-processing model (Chen et al. [9]) of face recognition, which represents the primary theoretical background of this research. Second, it contributes to social cognition frameworks which place specific emphasis on facial expression processing, a complex and critical component that clarifies the attentional dynamics that operate during emotion recognition tasks in non-clinical populations. In addition to focusing on accuracy, the present study emphasizes the mechanisms through which visual attention is distributed in accurately and inaccurately recognized emotions. 

## 2. Materials and Methods

### 2.1. Participants

A total of fifty-four participants were initially recruited through campus-wide announcements. Data from four participants (three females, one male) were excluded prior to analysis due to more than 5% missing gaze data. Based on previous research in eye-tracking studies, where a similar cutoff has been found to balance data loss with data integrity (e.g., [37,42]), a 5% missing data threshold was chosen, aligning with standard practices in the field while ensuring the reliability and robustness of the analyses. The final sample included fifty university students (24 female; 26 male) from various departments of a medium-sized private university. Ages of the participants ranged from 18 to 28 years (M = 23.23, SD = 2.23). The inclusion criteria included voluntary participation and successful eye-tracker calibration. The exclusion criteria included any past or current neurological or psychiatric diagnosis and any past or current use of psychotropic medication. All participants provided written informed consent prior to the experiment, and all completed the task. No identifiable personal information was collected. The study was approved by the Atılım University Review Board (approval no: ATÜ-LAP-C-1617-17:15.2.2017). All procedures were conducted in accordance with the ethical standards of the institutional and national research committees and with the 1964 Helsinki Declaration and its later amendments.

### 2.2. Materials and Apparatus

Stimulus presentation and data collection were controlled by a custom-built Windows application developed in C# (.NET Framework). The application presented colored photographs of male and female faces portraying five basic emotions—anger, disgust, fear, happiness, and sadness. For each emotion, one male and one female poser were included, yielding 10 unique experimental images. Inter-trial and inter-stimulus intervals were controlled with visual placeholders: a uniform gray screen and a centrally displayed “Next” prompt screen. Eye movements were recorded using a remote eye-tracking device (EyeTribe, The Eye Tribe Aps, Copenhagen, Denmark), operating at 60 Hz. The tracker was mounted centrally below the monitor and aligned with the participant’s gaze. The EyeTribe server software (C# SDK) handled calibration and real-time data streaming. Participants made forced-choice classifications by clicking one of five horizontally displayed emotion labels with a standard optical mouse.

### 2.3. Stimuli

All experimental stimuli were high-resolution, color photographs from the UC Davis Set of Emotion Expressions (UCDSEE) [42]. Each image depicted a poser displaying one of the five target emotions—anger, disgust, fear, happiness, or sadness—and was cropped to include only the head and upper shoulders against a neutral background. Each image measured 300 × 400 pixels on a 15.6-inch LCD display (1366 × 768 resolution; ~100.5 PPI), corresponding to a 7.25° × 9.62° visual angle at a 60 cm viewing distance.

Two additional image types were used to structure trial timing: blank screen: a uniform mid-gray square, identical in size to the face stimuli, displayed during inter-stimulus intervals; “Next” screen: the word “Next” presented centrally in a large sans-serif font, signaling the upcoming stimulus. For familiarization, participants completed a brief practice phase with four neutral facial expressions from the same poser set. These practice stimuli matched the experimental images in resolution and format but were excluded from the main task. The stimulus set included one male and one female poser per emotion, and this is a limitation of the present study. Although this design choice ensured strict control over stimulus AOIs, it inevitably restricted stimulus variability and generalizability.

### 2.4. Procedure

Two trained senior undergraduate research assistants, who were supported by the university’s faculty-led project through the Undergraduate Research Support Program, supervised the experiments. Participants were tested individually in a dimly lit, quiet laboratory. They were seated on an ergonomic office chair with a head and back rest, designed to stabilize the head at a distance of approximately 60 cm from the display. The session began with 9-point calibration of the EyeTribe tracker; calibration proceeded only if the mean error was below a 0.5° visual angle. This threshold was selected in line with established methodological standards in eye-tracking research, which recommend a 0.5° visual angle for ensuring accurate gaze-to-stimulus mapping in fine-grained analysis [41].

The experimental session consisted of a practice phase followed by the main trials. Each trial followed the same sequence: (1) Image display (2.0 s): A facial stimulus appeared at the screen center. (2) Blank screen (1.5 s): A uniform gray screen cleared the visual field. (3) Response screen (self-paced): Emotion label presentation and response collection were conducted at this screen. Five emotion word labels (anger, disgust, fear, happiness, and sadness) were displayed horizontally with a large sans-serif font; participants clicked the label that best matched the expression. (4) Inter-trial screen (1.0 s): The “Next” screen indicated the following trial. Stimuli were presented in a randomized order, and trial progression was fully automated by the software. For each trial, the stimulus identifiers, timestamps, classification decisions, gaze data (x–y coordinates), and fixation counts were logged into a structured text file. The full session, including calibration, practice, and experimental trials, lasted approximately 10–15 min.

**Figure 1:**
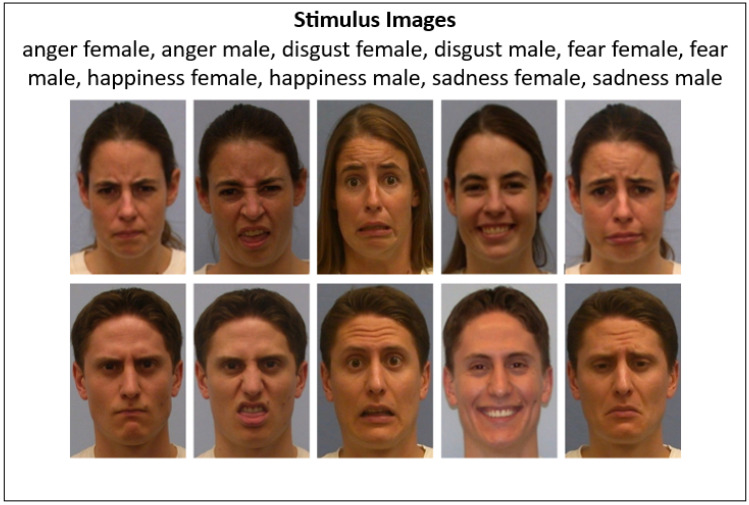
Diagram of the design and process of the study.

Areas of interest (AOIs) were defined for each face stimulus (300 × 400 px) using pixel coordinates. Table 1 presents the y-axis boundaries for the eye, nose, and mouth regions across male and female images of each emotion, ensuring consistent fixation mapping to relevant facial features. As can be seen in Figure 1, the stimulus images’ AOIs show variability in the mouth region. When the same poser expressed different emotions, the pixel position of the mouth region shifted. This natural variability in facial morphology across expressions contributed to differences in AOI boundaries (e.g., 269–391 pixels for the mouth).

### 2.5. Design

The study employed a within-subjects experimental design, where all participants completed classification tasks involving every emotion category. The independent variables were emotion (five levels: anger, disgust, fear, happiness, and sadness) and stimulus gender (two levels: male, female). The dependent variables included emotion classification accuracy (0–1), decision time, and eye movement measures (AOI fixation counts and gaze coordinates for each image-viewing period). The stimulus images are presented in Figure 1.

### 2.6. Data Preparation

All logged text data were transferred to SPSS data files by the researcher. Prior to statistical analyses, the gaze data were screened for completeness and quality. Following common practices in eye-tracking research [41], participants with more than 5% missing data for fixation measures were excluded from further analyses. This threshold was chosen to ensure sufficient data coverage across all experimental conditions and to reduce bias from incomplete gaze patterns. As a result, four participants (three females, one male) were removed, yielding a final sample of fifty for all reported analyses. All remaining data were inspected for outliers and normality; no additional cases were excluded. Statistical analyses were carried out based on 500 data points which were obtained for 10 stimulus images.

### 2.7. Statistical Analysis

**Figure 2:**
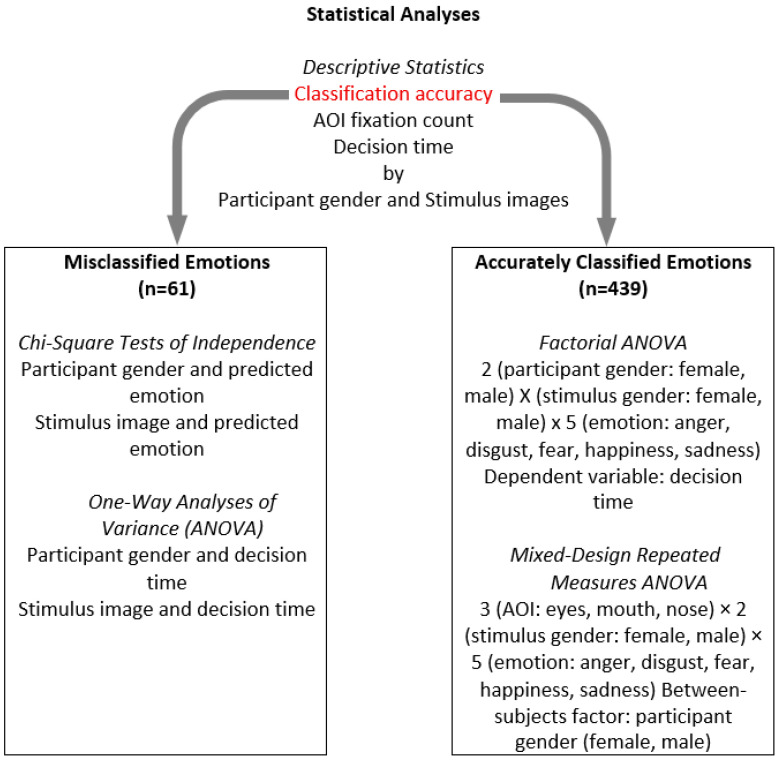
Overview of statistical analyses.

Prior to hypothesis testing, data were screened for statistical assumptions. Normality of residuals was assessed for each condition using Shapiro–Wilk tests, skewness/kurtosis values, and inspection of Q–Q plots. Sphericity was evaluated with Mauchly’s test; when violated (*p* < 0.05), Greenhouse–Geisser corrections were applied. For between-subject factors, homogeneity of variances was examined with Levene’s test. Outliers exceeding ±3 SD from the condition mean were inspected; none were removed beyond the pre-specified missing data exclusions described in the Section 2.6. The effect sizes are reported as the partial eta squared (η^2^_p_) for ANOVA effects. All pairwise comparisons were conducted using Bonferroni correction. Adjusted *p*-values for pairwise comparisons were reported. The data was analyzed using IBM SPSS.v.26. An overview of the statistical analysis is presented in Figure 2.

## 3. Results

### 3.1. Descriptive Statistics

Preliminary analyses were conducted with the entire dataset (500 data points). The descriptive statistics of the study variables by participant gender are presented in Table 2.

Classification analyses were carried out in two parts. The first part examined the patterns of the inaccurate classification of emotions, and the second part explored the patterns of accurate emotion predictions. In this direction the dataset was separated by the emotion classification accuracy: of the 500 data points, 439 (87.8%) of the predictions were accurate and 61 (12.2%) were inaccurate.

### 3.2. Patterns in Inaccurate Facial Emotion Recognition

To examine the pattern of inaccurately classified emotions, first, descriptive statistical analyses were conducted. A Chi-Square Test of Independence was conducted to determine whether there were significant associations between participants’ gender and the inaccurately classified emotions. The results indicated a non-significant association between gender and misclassified emotions *X*^2^(6) = 6.515, *p* = 0.368.

**Figure 3:**
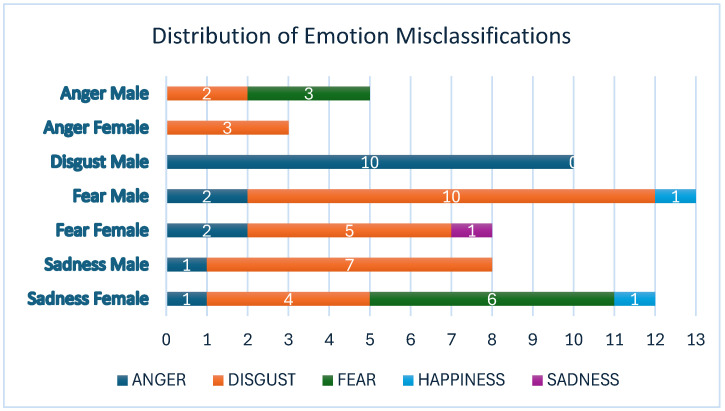
Distribution of emotion misclassifications. Note: vertical axis: stimulus image; horizontal axis: misclassified emotion.

A second Chi-Square Test was conducted to determine whether the emotion in stimulus images was systematically misclassified as particular emotions. The test result showed a significant association (*X*^2^(24) = 67.206, *p* = 0.036). As Figure 1 shows, fear male was the most frequently misclassified image. It was mostly misclassified as disgust (10 participants) and anger (2 participants). Disgust male, on the other hand, was misclassified exclusively as anger (10 participants). The second most frequently misclassified image was sadness female, which was misclassified as fear (six participants) and disgust (four participants). The distribution of emotion misclassifications is presented in Figure 3. As can be seen in Figure 3, fear male and sadness female stimulus images were the most frequently misclassified emotions. Disgust emerged as the most frequently assigned predicted emotion, particularly when the stimulus image expressed fear, anger, or sadness.

To assess the suitability of the data for subsequent parametric analyses, Shapiro–Wilk tests were performed. The results indicated that decision time was approximately normally distributed (*W* = 0.945, *p* = 0.027), whereas AOI fixation counts for the eyes (*W* = 0.711, *p* < 0.001), mouth (*W* = 0.789, *p* < 0.001), and nose (*W* = 0.573, *p* < 0.001) deviated from normality.

Stimulus images with fewer than five responses were removed from the analysis. The final dataset for follow-up analyses included five stimulus images: disgust male, fear female, fear male, sadness female, and sadness male. Power analysis using G*Power 3.1.9.7 indicated that the sample size met the requirements for *F* tests (1–β = 0.95).

A series of One-Way ANOVAs were conducted to examine differences in decision time across stimulus images and participant gender. The first one-Way ANOVA, which was conducted to determine if decision time varies by participant gender, indicated no significant difference in decision time between males (M = 7828.32, SD = 3902.88, 95% CI [6312.11–8845,53]) and females (M = 6278.88, SD = 3489.32, 95% CI [4568.16–7318.49]) (*F*(51) = 2.238, *p* = 0.141, η^2^ = 0.049). Similarly the second one-Way ANOVA indicated no significant differences in decision time between the five stimulus conditions (*F*(57) = 0.251, *p* = 0.908, η^2^ = 0.089): disgust male (M = 7227.40, SD = 3764.33, 95% CI [4821.63–9633.17]), fear male (M = 7376.85, SD = 3181.63, 95% CI [5266.85–9486.85]), fear female (M = 7022.88, SD = 2659.00, 95% CI [4333.14–9712.61]), sadness male (M = 7646.38, SD = 3252.73, 95% CI [4956.64–10336.11]) and sadness female (M = 6119.33, SD = 5334.80, 95% CI [3923.18–8315.49]).

#### Fixation Distributions for the Misclassified Stimulus Images

**Figure 4:**
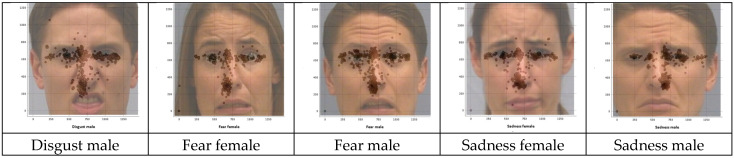
Fixation distributions for the misclassified stimulus images.

Finally, the fixation distributions for the misclassified stimulus images are presented in Figure 4. Across all five panels, hotspots align tightly with the eye and nose AOIs defined in Table 1, and rarely spill below the upper -mouth boundary, indicating insufficient lower-face sampling during the trials, which produced incorrect responses. Poser gender did not qualitatively alter the pattern: female faces showed a slightly broader eyes AOI spread, whereas male faces showed tighter central clustering, but both exhibited low mouth AOI coverage.

In summary, inaccurate recognition was not influenced by participant gender but showed systematic patterns across emotions; in particular, fear male and sadness female were most frequently misclassified, most often as disgust or anger. Disgust emerged as the dominant predicted emotion, suggesting perceptual overlap among negatively valenced expressions. Misclassified trials were further characterized by restricted fixation on the eyes and nose, with limited mouth sampling, highlighting insufficient integration of lower-face cues as a likely contributor to these systematic errors.

### 3.3. Patterns in Accurate Facial Emotion Recognition

#### 3.3.1. Decision Time

Mauchly’s test of sphericity was significant for the emotion variable (W = 0.113, *p* < 0.001); therefore, Greenhouse–Geisser correction was applied (Ɛ = 0.548). To examine the difference in decision time, a 2 (participant gender: female, male) × (stimulus gender: female, male) × 5 (emotion: anger, disgust, fear, happiness, sadness) factorial ANOVA was conducted. The analysis revealed a main effect of emotion (*F*(4412) = 11.771 *p* < 0.001, η^2^ = 0.10) and participant gender (*F*(1412) = 4.573, *p* = 0.033, η^2^ = 0.0), as well as a significant emotion xX participant gender interaction (*F*(1412) = 4.858 *p* = 0.001, η^2^ = 0.05). Pairwise comparisons using Bonferroni correction revealed that happiness had the shortest decision time and was classified statistically faster than fear (M = 5597.78, SE = 205.77, 95% CI [5193.28, 6002.28], *p* < 0.001) and anger (M = 6132.97, SE = 190.40, 95% CI [5758.68, 6507.25] *p* < 0.001). Anger, on the other hand, had the longest decision time, and was classified slower than sadness (M = 5019.03, SE = 204.69, 95% CI [4616.66, 5421.39], *p* = 0.001) and disgust (M = 4935.17, SE = 193.92, 95% CI [4554.01, 5316.39], *p* < 0.001). The differences between other emotion pairs did not reach significance. The mean estimates of decision time by emotion are presented in Table 3.

As for the main effect of participant gender, female participants’ decision time (M = 5037.90, SE = 125.46, CI [4792.89–5288.27]) was shorter than male participants’ decision time (M = 5411.83, SE = 121.37, CI [5176.06–5653.63], *p* = 0.033).

**Figure 5:**
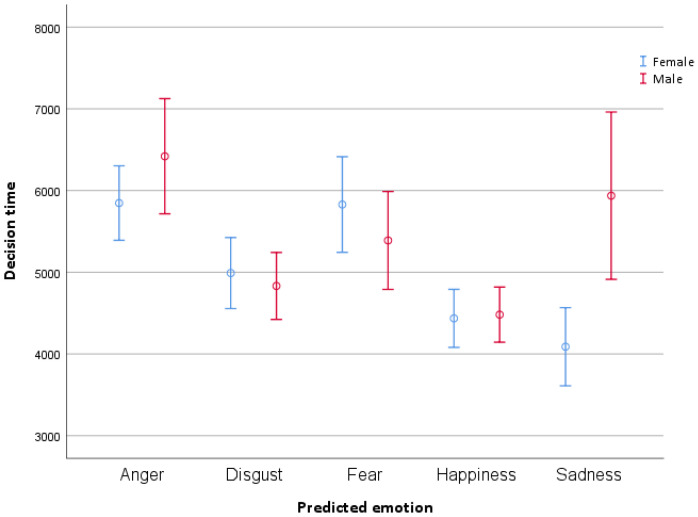
Graphical display of mean estimates of decision time by emotion and participant gender.

The emotion × participant gender interaction revealed that sadness was the only emotion showing a significant gender difference in decision time. Females (M = 4100.95, SE = 293.36, 95% CI [3524.28–4677.62]) responded faster than males (M = 5937.10, SE = 285.53, 95% CI [5375.81–6498.39], *p* < 0.001). The mean estimates and standard errors by emotion and participant gender are presented in Table 4. Figure 5 provides a graphical display of decision times with 95% confidence intervals.

#### 3.3.2. AOI Fixation Count

A 3 (AOI: eyes, mouth, nose) × 2 (photo gender: female, male) × 5 (emotion: anger, disgust, fear, happiness, sadness) mixed-design repeated-measures ANOVA was conducted, with participant gender (female, male) as a between-subjects factor. The main effect of participant gender approached significance (*F*(1, 48) = 3.92, *p* = 0.054, η^2^_p_ = 0.075), with males (M = 60.49, SE = 3.25, 95% CI [53.97–67.02]) tending to fixate longer overall than females (M = 51.22, SE = 3.38, 95% CI [44.43–58.01]).

##### Main Effects

The multivariate test yielded a significant main effect of *emotion* (Wilks’ Λ = 0.375, *F*(4, 45) = 18.78, *p* < 0.001, η^2^_p_ = 0.625). Bonferroni-adjusted pairwise comparisons indicated that the fixation count for happiness (M = 44.75, SE = 1.57, 95% CI [41.59, 47.92]) was significantly lower than for all other emotions. Disgust counts (M = 50.08, SE = 1.96, 95% CI [46.14–54.02]) were significantly lower than anger (M = 64.22, SE = 2.54, 95% CI [59.12–69.33]) and fear (M = 64.65, SE = 5.68, 95% CI [53.2–76.07] counts. No other emotion comparisons reached significance. The Bonferroni-adjusted pairwise comparison of mean estimates of emotion pairs is presented in Table 5.

The main effect of stimulus image gender was not significant (Wilks’ Λ = 0.998, *F*(1, 48) = 0.11, *p* = 0.741, η^2^_p_ = 0.002). The multivariate test indicated a significant main effect of AOI as well (Wilks’ Λ = 0.087, *F*(2, 47) = 247.95, *p* < 0.001, η^2^_p_ = 0.913), indicating that fixation counts varied markedly across facial regions. Bonferroni-adjusted pairwise comparisons revealed that participants looked at the eyes (M = 105.08, SE = 4.24, 95% CI [96.56–113.61]) significantly longer than the mouth (M = 57.23, SE = 3.86, 95% CI [49.46–64.99], *p* < 0.001), and both were viewed longer than the nose (M = 5.25, SE = 0.76, 95% CI [3.73–6.78], *p* < 0.001).

##### Interactions

The AOI Fixation count *×* emotion interaction was significant (Wilks’ Λ = 0.302, *F*(8, 41) = 11.82, *p* < 0.001, η^2^_p_ = 0.698), indicating that the number of fixations across emotions varied by face region. Other interactions were not statistically significant. The significant differences by AOIs are presented below.

Eyes: The fixation count for anger (M = 128.47, SE = 7.82, 95% CI [118.78–138.15]) was higher than for happiness (M = 92.21, SE = 4.87, 95% CI [82.42, 102.00], *p* < 0.001), disgust (M = 94.68, SE = 3.64, 95% CI [87.37, 101.99], *p* < 0.001), and sadness (M = 98.12, SE = 7.39, 95% CI [83.25, 112.98], *p* < 0.001). Fear (M = 111.94, SE = 7.82, 95% CI [96.22, 127.66]) was not significantly different from any other emotion other than anger. The difference in the eye region fixation counts did not reach significance for any other emotion pairs. Means, standard errors, and 95% confidence intervals of AOI fixation counts by emotion × AOI are presented in Table 6.

Mouth: As Table 6 illustrates, in the mouth area, happiness had the lowest count of fixations (M = 38.26, SE = 3.39, 95% CI [31.44–45.08], *p* < 0.001), significantly lower than all other emotions (anger, fear, disgust and sadness). The only other significant difference in fixation counts in mouth area was between fear (M = 75.95, SE = 9.57, 95% CI [56.70–95.20]) and disgust (M = 51.27, SE = 3.36, 95% CI [43.33–59.25]). The difference in the mouth area fixation counts did not reach significance in any other emotion pairs.

**Figure 6:**
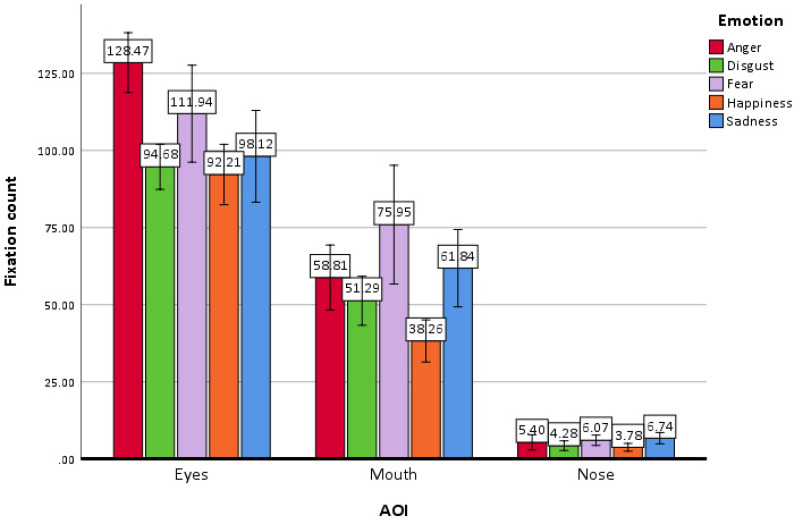
Fixation counts by AOIs.

Nose: In the nose area, happiness elicited the fewest fixations (M = 3.78, SE = 0.625, 95% CI [2.53–5.04]), significantly fewer than fear (M = 6.07, SE = 0.83, 95% CI [4.40–7.75], *p* = 0.008) and sadness (M = 6.74, SE = 0.92, 95% CI [4.89–8.59], *p* < 0.001). Sadness received the highest fixations aside from happiness (*p* < 0.001), significantly differing only from disgust (M = 4.28, SE = 0.78, 95% CI [2.72–5.84], *p* = 0.009) aside from Happiness (*p* < 0.001). The difference in the mouth area fixation counts did not reach significance in any other emotion pairs. A graphic representation of the fixation counts by AOIs is presented in Figure 6.

##### Fixation Distributions for the Stimulus Images

**Figure 7:**
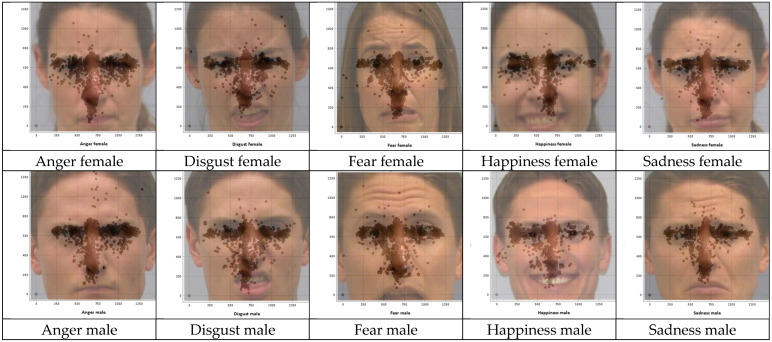
Fixation distributions for the accurately classified stimulus images.

Figure 7 presents heatmaps of the fixation distributions, where darker regions indicate higher fixation density. Across emotions, fixations were predominantly concentrated in the eyes and nose AOIs, with increased mouth AOI fixations for happiness and increased nose AOI fixations for disgust.

### 3.4. Comparison of AOI Fixation Distributions for Misclassified Stimulus Images

When the AOI fixation distributions of misclassified stimulus images (disgust male, fear female, fear male, sadness female and sadness male) were visually examined and compared with the fixation distributions of the same stimulus images when accurately recognized, some differences were observed. A comparison of the fixation distributions of stimulus images for misclassified and accurately recognized trials is presented below.

Disgust male: Misclassified trials for this stimulus image had fixations concentrated in the eyes AOI, with a narrow streak along the nasal bridge and sparse sampling of the mouth AOI, whereas accurate trials preserved eyes AOI sampling and added broader nose AOI coverage with increased fixations to the upper-lip region, consistent with use of the nose-wrinkle/upper-lip-raise cues.

Fear female: Misclassified trials for this stimulus image showed dominant fixation on the eyes AOI and limited exploration beyond the nose AOI, with minimal mouth AOI sampling, whereas accurate trials maintained strong eyes AOI sampling but expanded into the nose and mouth AOIs, consistent with use of the open-mouth diagnostic cue.

Fear male: Misclassified trials for this stimulus image exhibited dense eyes AOI fixations and a narrow nose AOI streak with little coverage of the mouth AOI; in contrast, accurate trials added broader nose AOI sampling and substantially more fixations to the mouth, leveraging the open-mouth cue.

Sadness female: Misclassified trials for this stimulus image focused on the eyes AOI with a marked thin nose AOI along the nasal-bridge column and limited coverage of the mouth AOI along mouth corners, whereas accurate trials retained eyes AOI sampling and increased fixations across the nose AOI and mouth AOI across lip corners, consistent with the downturned-mouth cue.

Sadness male: Misclassified trials for this stimulus image were eyes AOI-dominant with sparse mouth AOI sampling; accurate trials added broader nose AOI coverage and more mouth AOI fixations across lip corners, consistent with reliance on lower-face diagnostics. Taken together, these comparisons reveal consistent differences between fixation patterns for accurately recognized versus misclassified expressions.

In summary, across emotions, misclassified trials showed a restricted fixation patterns dominated by the eyes, whereas accurate recognition was associated with broader sampling that incorporated diagnostic cues from the nose and mouth AOIs. This suggests that recognition accuracy depends on integrative use of both upper and lower facial features.

## 4. Discussion

The present study employed a within-subjects experimental design incorporating eye-tracking methodology to examine the patterns of accurate and inaccurate recognition of basic facial emotions, with particular emphasis on the roles of both participant gender and the gender of the emotional stimulus. By simultaneously investigating five basic emotions (anger, disgust, fear, happiness, and sadness) in young healthy adults, this study extends the existing literature on facial emotion recognition and visual attention to emotional cues in the three main areas of the face. This design enabled the simultaneous examination of both accurate and inaccurate recognition. By doing so, the study moves beyond traditional accuracy-based approaches and highlights attentional dynamics underlying misclassification, offering a novel contribution to theories of social cognition.

The findings of this study can be interpreted within the context of recent evidence regarding the variability of fixation patterns in emotion recognition. While studies by Yitzhak et al. [21] and Paparelli et al. [22] have compellingly demonstrated that successful recognition can be achieved through diverse, idiosyncratic gaze patterns across individuals, the current study shifts the focus to the systematic nature of misclassification. In doing so, the results of the present study both challenge and extend this perspective.

### 4.1. Inaccurate Recognition

No significant association was found between participant gender and misclassification patterns, which is consistent with the extant literature supporting the absence of gender differences in the recognition of explicit facial expressions [43,44]. This finding appears robust, suggesting that misclassification mechanisms may be largely invariant across genders, at least within young healthy samples. Consistent with prior research suggesting that negative emotions are more susceptible to misrecognition [23,43], the findings of this study revealed systematic patterns of misclassification, particularly for fear and sadness. Notably, fear expressed on male faces was the most frequently misclassified emotion, most often perceived as disgust. This finding aligns with earlier reports that fear expressions were recognized significantly less accurately than other emotions [43,45] and shared similar recognition accuracy rates [46], as well as perceptual features, with disgust [47], making them particularly difficult to differentiate. This replicates the well-established difficulty in differentiating fear from disgust and strengthens confidence that this bias reflects perceptual overlap rather than sample-specific noise.

Similarly, sadness displayed on female faces was frequently interpreted as fear or disgust, a tendency consistent with previous work [43], indicating that sadness is often confused with other negative emotions, especially when expression intensity is low [48]. This consistent difficulty in accurate recognition of negative emotions may also be related to configural versus featural information processing during facial emotion recognition, which suggests that processing style depends on the expressed emotion [49]. Bombari et al. [45], for example, reported that recognition of fear relies more on featural information, while configural processing dominates the recognition of sadness. The misclassification of sadness in the present study should be interpreted more cautiously, however, as intensity cues and the limited stimulus set may have amplified this tendency. Disgust emerged as the most frequently assigned emotion in cases of misrecognition, particularly when the target expression was fear, anger, or sadness. This finding aligns with the results obtained in Poncet and colleagues’ [23] experiments, where recognizing disgust in other expressions was significantly more frequent. This tendency may reflect the perceptual salience of disgust-related features, such as nose wrinkling or upper lip retraction, which can be visually dominant and easily over-attributed [50]. Du et al. [47], for instance, indicated that it is possible to have compound emotions with shared expressive features, such as being “fearfully disgusted” or “angrily disgusted,” in which facial action units combine features of fear and disgust or anger and disgust, respectively. Alternatively, this misclassification bias may suggest an adaptive perceptual strategy [51], as disgust signals contamination and health risks and may thus be evolutionarily prioritized in ambiguous contexts [52].

Participant gender did not significantly influence patterns of misclassification, converging with findings that the perceptual mechanisms underlying misrecognition are largely invariant across genders [45]. Moreover, decision times did not differ significantly for incorrect classifications, implying that errors were not due to hesitation or uncertainty, but rather to biologically based [53], automatic, or complex emotional and cognitive processing. This interpretation is consistent with current state-of-the-art perspectives in emotion processing, which indicate substantial overlap between brain systems underlying emotion recognition [49]. The results of eye-tracking data extend the work of Poncet et al. [23] by revealing the specific attentional mechanisms underlying these systematic errors: misclassifications were characterized by a failure to adequately sample diagnostic features (e.g., the nose region for disgust), leading their model to predict inaccurate recognitions. Together, by treating errors as structured outcomes rather than statistical noise, these patterns indicate that misclassification is systematic and perceptually grounded. This finding particularly contributes to face-processing theories where the first stage of information processing involves encoding the features, and the second involves processing of social information, i.e., facial expression and eye gaze.

### 4.2. Accurate Recognition

Happiness was, as expected, the most accurately and rapidly recognized emotion, replicating a well-established finding in the literature [2,43,45,46,54]. This convergence underscores that happiness recognition constitutes a reliable benchmark against which more variable recognition patterns can be contrasted. Happiness also elicited the fewest fixations across all areas of interest (AOIs), suggesting high efficiency in processing positive affect, potentially due to its distinct and universally recognized facial features. Consistent with the heatmaps, happiness uniquely elicited increased mouth AOI sampling while maintaining high eye AOI sampling. Importantly, in the present study, happiness was the only facial expression for which the mouth displayed exposed teeth, indicating the critical importance of controlling mouth display in future facial emotion expression studies [23,55].

In contrast, anger, though generally recognized with high accuracy, required the longest decision times and prompted the highest number of fixations, especially in the eye region. This result contradicts the findings of Poncet et al. [23], who reported more accuracy in recognition of anger when fixated on the mouth area compared to the eye area. This is consistent with the view that anger demands enhanced cognitive and visual scrutiny, possibly due to its social relevance and threat-related implications [56]. Importantly, anger expressions are also known to be interpreted more accurately in female than in male faces, which may reflect cultural stereotypes about gendered emotional expressivity [48]. Fear and anger share common underlying characteristics, as both are negatively valenced, associated with high arousal and signal threat [56]. This effect appears robust, aligning with theoretical accounts that anger demands enhanced cognitive and visual scrutiny due to its social relevance and threat potential.

Gender differences were observed in decision times, with female participants responding more quickly than males. This aligns with previous research suggesting superior emotion recognition speed and sensitivity among women [44,51,54]. The only significant gender × emotion interaction was for sadness, where females responded significantly faster, possibly reflecting gendered emotional socialization patterns [57], intentions [58], or greater accuracy in responding to both target and non-target sadness cues [59]. However, this finding in the present study should be considered exploratory, as the limited stimulus set and modest sample size restricts the strength of this conclusion.

### 4.3. Eye-Tracking Patterns and AOI-Specific Fixation Trends

Eye-tracking results provided further insight into the attentional mechanisms underlying emotion recognition. The eyes were the most focused-on region across emotions, consistent with earlier eye-tracking studies with healthy individuals [22,44] and reinforcing the centrality of ocular cues in both encoding [60] and face-recognition performance [61].

Fixations on the eyes, particularly for anger and fear, confirm their status as the most diagnostically rich facial features [40,46]. Anger elicited the highest fixations in the eye region, while fear led to the greatest visual attention to the mouth, potentially due to the open-mouth configuration typical of fear expressions [47]. High fixation on the mouth suggests that this region may carry critical but not always sufficient cues, especially in combination with distinctive eyebrow cues, which are often among the least accurately recognized [25]. Eye-tracking studies have also supported these results for fearful expressions, with attention directed toward both the eyes and mouth [22,62].

Interestingly, in contrast to other findings reporting more eye area fixation [23], sadness elicited the highest fixation counts in the nose area, which in this study also included the nasolabial region, a relatively underexplored AOI in emotion research. This may suggest that observers also rely on subtle musculature changes around the nose to differentiate sadness from other negative expressions. Prior work has largely overlooked the diagnostic role of the nose region, but emerging evidence suggests it may carry disambiguating micro-features for low-intensity expressions [63]. A recent study using convolutional neural networks revealed the importance of features around the nose, showing that features in the nose and mouth areas serve as critical landmarks for neural networks [64]. Given the paucity of prior work on nose AOI, this result should be treated as preliminary. AOI fixation distribution heatmaps also supported quantitative results, indicating accuracy differences: when trials included incremental sampling of the nose AOI, recognition improved; when sampling remained confined to the eye AOI, errors increased. Put differently, accurate trials reflect dual-region evidence integration (eyes plus nose/mouth AOI), whereas misclassifications reflect eye AOI reliance only.

Overall, these findings support the notion that visual attention patterns vary systematically across basic emotions, and that different emotions evoke distinct AOIs [64,65]. However, facial expressions of emotion cannot be reduced to simple feature processing [63]. Underlying individual differences in face recognition ability [61] may also play an important role, and further studies are needed to better understand nuances in the recognition of basic emotions. Beyond the statistical findings, fixation distributions provide compelling visual confirmation of attentional biases across emotions and stimulus genders. Importantly, the salience of underexplored AOIs such as the nose underscores the need to expand conventional models of facial emotion recognition, which have traditionally prioritized the eyes and mouth. The results of the current study present a nuanced picture in the debate on fixation patterns. While there was considerable individual variability in scanning paths for correctly identified emotions, supporting the idea of Yitzhak and colleagues [21], the findings challenge a strong interpretation of pure idiosyncrasy by demonstrating that specific misclassifications are associated with more systematic and constrained patterns of attention (e.g., under-sampling the nose). This indicates that while the pathways to success may be many and idiosyncratic [22], the pathways to specific errors might be fewer and more stimulus-driven [23]. The balance of converging and novel evidence in this research underscores the value of integrating misclassification and gaze analysis in a multimodal approach to advance information-processing and social cognition theories of emotion perception.

#### 4.3.1. Limitations and Future Directions

While this study contributes novel insights, it has several limitations. First, the use of static facial images that included only one male and one female poser per emotion may limit ecological validity; dynamic expressions might elicit different recognition and fixation patterns. However, a recent finding indicates that idiosyncratic fixation patterns generalize across dynamic and static stimuli [22], which suggests that the core classification mechanisms that this study found might be robust. Second, this study focused solely on basic emotions; future research should explore complex or self-conscious emotions, which may involve different cognitive and visual mechanisms. Additionally, incorporating contextual variables could shed light on recognition of basic emotions and eye-tracking behavior. Finally, although participant and stimulus gender were considered, factors such as emotional intensity and individual empathy levels were not assessed and could influence recognition patterns. As a result, the findings may not generalize across a broader range of facial expressions.

#### 4.3.2. Implications

The present findings provide both robust and exploratory contributions that extend existing theories of facial emotion recognition and social cognition while also offering implications for understanding emotion recognition in real-world interpersonal contexts. From a theoretical standpoint, the results help refine basic-emotion theory [1,5] by showing that recognition errors, particularly for fear and disgust, follow systematic patterns and may increase the possibility of challenging the assumption that all basic emotions are equally distinct. The findings also enrich two-stage information-processing models of facial emotion recognition [45] by showing that happiness can be identified rapidly with minimal fixation, whereas some emotions remain vulnerable to confusion at the configural stage, with exploratory evidence pointing to the diagnostic value of underexamined facial features. Moreover, the results of this study engage with contemporary debates by demonstrating that systematic errors exist within a system that also allows for successful idiosyncratic recognition [21,22], thereby challenging a purely uniform view of scanning strategies and highlighting the need for models that account for both successful and unsuccessful outcomes. Within social cognition models of facial expressions [7] are key cues for interpreting others’ intentions and guiding interpersonal behavior. The present study shows that systematic misclassifications, such as perceiving fear as disgust, are not random but structured, which implies that social cognitive inferences may be consistently biased by perceptual overlap. The systematic misclassifications observed in this study, particularly the tendency to confuse fear and sadness with disgust, highlight how perceptual biases may distort emotional communication in everyday interactions. Misinterpretation of fear as disgust, for instance, may lead to inappropriate social responses, such as perceiving vulnerability as rejection or hostility, thereby potentially disrupting interpersonal trust and empathy. Similarly, misrecognizing sadness may reduce opportunities for providing social support, a key protective factor in psychological well-being.

The second real world implication of the results directly relates to the efficiency in interpersonal emotion recognition. The findings reinforce the importance of visual attention mechanisms in shaping emotion decoding accuracy. That participants relied heavily on the eye and mouth regions, with sadness uniquely eliciting increased fixation on the nose, suggests that attentional strategies differ across emotions and may affect the efficiency of interpersonal emotion recognition.

Gender-related differences, such as women’s faster responses to sadness, may reflect broader patterns of emotional socialization and empathic attunement. These patterns may translate into gendered differences in relationship dynamics, where women often assume roles requiring higher emotional sensitivity. Importantly, the absence of strong participant-gender effects on overall accuracy suggests that the basic perceptual mechanisms of facial emotion recognition are robust across genders, but nuanced socialization differences may emerge in the speed and style of processing.

From a clinical perspective, these findings are relevant both to populations with known emotion recognition deficits and to healthy young individuals. The present evidence that certain emotions (fear, sadness) are more vulnerable to inaccurate recognition, and that distinct AOIs carry diagnostic value, may inform targeted training programs designed to enhance emotion recognition skills through eye-tracking-based feedback.

Finally, these results have implications for applied settings such as human–computer interaction and affective computing. Emotionally responsive technologies, including social robots and adaptive educational software, increasingly rely on accurate modeling of human emotional perception [66]. Understanding which features are most visually diagnostic across different emotions and which expressions are most prone to misclassification can guide the development of more effective artificial systems that interact seamlessly with human users.

## 5. Conclusions

This study enhances our understanding of the visual–cognitive mechanisms underlying facial emotion recognition, highlighting both emotion-specific and limited gender-related effects. Of special importance, the study examined accurate- and inaccurate-recognition processes separately, rather than relying solely on proportional accuracy, which may jeopardize the validity by increasing Type I error [3]. The findings suggest that while happiness is processed quickly and efficiently, negative emotions, particularly fear and disgust, are more susceptible to misrecognition and require increased visual scrutiny, especially in emotionally ambiguous contexts. Taken together with the AOI fixation distribution contrasts, these findings suggest that the scanpath strategy, especially sampling beyond the eyes into the nose and mouth AOIs, underlies the accuracy of basic-emotion recognition. These results have important implications for theories of emotion processing, social cognition, and applied fields such as clinical psychology and human–computer interaction.

## Figures and Tables

**Table 1:** AOI Coordinates (in pixels) for eyes, nose, and mouth region stimulus images (image size = 300 × 400 px).

Stimulus Images	Eyes (y)	Nose (y)	Mouth Start (y)	Mouth End (y)
Anger Female	239	275	315	353
Anger Male	258	297	337	379
Disgust Female	247	284	317	366
Disgust Male	216	250	286	334
Fear Female	213	248	269	358
Fear Male	217	277	312	373
Happiness Male	228	275	315	370
Happiness Female	241	286	316	359
Sadness Female	252	294	336	373
Sadness Male	258	301	343	391

**Table 2:** Descriptive statistics of the study variables by participant gender (*N* = 50).

	AOI Fixation Count									Classification Task					
	Eyes			Mouth			Nose			Classification Accuracy			Decision Time (s)		
Stimulus image	Female M SD	Male M SD	Total M SD	Female M SD	Male M SD	Total M SD	Female M SD	Male M SD	Total M SD	Female M SD	Male M SD	Total M SD	Female M SD	Male M SD	Total M SD
Anger male	108.71 38.2	155.36 65.83	134.07 59.26	38.81 19.90	59.92 38.7	50.28 32.98	5.33 11.54	6.68 6.45	6.07 9.05	0.95 0.22	0.84 0.37	0.89 0.31	5.18 1.51	6.59 2.38	5.94 2.13
Anger female	127.86 38.9	135.92 41.37	132.24 40.03	65.33 59.53	70.52 65.30	68.15 62.1	4.95 14.54	4.92 5.46	4.93 10.48	1 0.00	0.88 0.33	0.93 0.25	6.68 2.08	6.77 2.72	6.73 2.42
Disgust male	99.71 48.34	124.36 120.37	113.11 94.45	60.05 48.48	66.92 80.68	63.78 67.29	4.33 6.26	6.2 10.87	5.35 9.02	0.71 0.46	0.84 0.37	0.78 0.42	5.69 2.75	5.64 2.11	5.67 2.4
Disgust female	85.71 34.36	103.08 46.22	95.15 41.72	42.52 27.46	53.16 54.15	48.3 43.9	3.67 4.84	4.48 7.93	4.11 6.64	0.95 0.22	1 0.00	0.98 0.15	4.56 1.49	4.99 2.22	4.79 1.91
Fear male	101.76 45.36	140.36 96.6	122.74 79.18	56.19 33.35	102.04 118.12	81.11 92.02	3.29 2.94	8.48 9.43	6.11 7.62	0.67 0.48	0.80 0.41	0.74 0.44	5.44 2.22	6.87 3.33	6.22 2.94
Fear female	110.67 57.36	101.88 103.26	105.89 84.67	70.86 32.49	76.8 93.90	74.09 71.98	5.71 11.1	4.76 4.75	5.2 8.19	0.86 0.36	0.80 0.41	0.83 0.38	6.2 2.07	5.51 2.19	5.82 2.14
Happiness male	89.76 41.12	90.92 25.2	90.39 33.02	41.71 37.38	38.64 46.31	40.04 42.04	3.24 4.3	4.28 4.73	3.8 4.52	1 0.00	0.96 0.20	0.98 0.15	4.58 1.61	4.34 1.06	4.45 1.33
Happiness female	80.52 36	106.84 59.82	94.83 51.57	40.67 29.33	40.84 21.73	40.76 25.19	3.14 6.07	4.28 5.34	3.76 5.65	1 0.00	1 0.00	1 0.00	4.05 0.87	4.81 1.51	4.47 1.3
Sadness male	70.29 31.52	130 124.23	102.74 97.86	44.71 20.38	82.04 78.87	65 62.09	5.67 6.51	7.76 8.54	6.8 7.67	0.90 0.30	0.84 0.37	0.87 0.34	4.19 1.86	6.44 3.22	5.41 2.89
Sadness female	72.67 38.42	119.36 73.07	98.04 63.69	50.57 33.55	76.44 93.15	64.63 72.78	6.05 9.64	6.24 8.35	6.15 8.86	0.76 0.44	0.76 0.44	0.76 0.43	4.42 2.12	6.28 4.39	5.43 3.63
Total	94.77 44.25	120.81 83.00	108.92 69.26	51.14 36.9	66.73 75.41	59.62 61.37	4.54 8.43	5.81 7.47	5.23 7.94	0.88 0.32	0.87 0.33	0.88 0.33	5.10 2.06	5.82 2.76	5.49 2.49

**Table 3:** Mean estimates and standard errors of decision time by emotion.

Emotion	M	SE	95% CI (LL–UL)
Anger	6132.97	190.40	(5758.68–6507.25)
Disgust	4935.17	193.92	(4554.01–5316.39)
Fear	5597.78	205.77	(5193.28–6002.28)
Happiness	4453.60	182.61	(4094.63–4812.57)
Sadness	5019.03	204.69	(4616.66–5421.39)

**Table 4:** Mean estimates and standard errors of decision time by emotion and participant Gender.

Emotion	Participant Gender	M	SE	95% CI (LL–UL)
Anger	Female	5846.44	266.26	(5323.03–6369.84)
	Male	6419.50	272.25	(5884.33–6954.67)
Disgust	Female	5022.77	284.15	(4464.20–5581.34)
	Male	4847.62	263.95	(4328.76–5366.49)
Fear	Female	5800.52	299.64	(5211.51–6389.52)
	Male	5395.06	282.12	(4840.48–5949.61)
Happiness	Female	4432.24	263.48	(3914.31–4950.16)
	Male	4474.97	252.92	(3977.78–4972.14)
Sadness	Female	4100.95	293.36	(3524.28–4677.62)
	Male	5937.10	285.53	(5375.81–6498.39)

**Table 5:** Bonferroni-adjusted pairwise comparison of mean estimates of AOI fixation count by emotion pairs.

Emotion 1	Emotion 2	M Difference	SE Difference	95% CI (LL–UL)	*p*
Anger (64.23, 2.54)	Disgust	14.14	2.90	(5.60–22.68)	<0.001
	Fear	−0.43	5.88	(−17.74–16.87)	1.000
	Happiness	19.47	2.60	(11.83–27.12)	<0.001
	Sadness	8.66	4.83	(−5.57–22.88)	0.796
Disgust (50.08, 1.96)	Anger	−14.14	2.90	(−22.68–5.60)	<0.001
	Fear	−14.57	4.70	(−28.40–0.75)	0.032
	Happiness	5.33	1.77	(0.12–10.54)	0.042
	Sadness	−5.48	3.10	(−14.59–3.62)	0.828
Fear (64.65, 5.68)	Anger	0.43	5.88	(−16.87–17.74)	1.000
	Disgust	14.57	4.70	(0.75–28.40)	0.032
	Happiness	19.90	5.47	(3.82–35.99)	0.007
	Sadness	9.09	4.83	(−5.13–23.31)	0.660
Happiness (44.75, 1.58)	Anger	−19.47	2.60	(−27.12–11.83)	<0.001
	Disgust	−5.33	1.77	(−10.54–0.12)	0.042
	Fear	−19.90	5.47	(−35.99–3.82)	0.007
	Sadness	−10.82	3.58	(−21.35–0.28)	0.040
Sadness (55.57, 4.15)	Anger	−8.66	4.83	(−22.88–5.57)	0.796
	Disgust	5.48	3.10	(−3.62–14.59)	0.828
	Fear	−9.09	4.83	(−23.31–5.13)	0.660
	Happiness	10.82	3.58	(0.28–21.35)	0.040

**Table 6:** Means, standard errors, and 95% confidence intervals of fixation counts by Emotion × AOI fixation count.

	AOI								
Emotion	Eyes			Mouth			Nose		
	M	SE	95% CI	M	SE	95% CI	M	SE	95% CI
Anger	128.47	4.82	(118.78–138.15)	58.81	5.24	(48.28–69.34)	5.40	1.23	(2.93–7.86)
Disgust	94.68	3.64	(87.37–101.99)	51.29	3.96	(43.33–59.25)	4.28	0.78	(2.72–5.84)
Fear	111.94	7.82	(96.22–127.66)	75.95	9.57	(56.70–95.20)	6.07	0.83	(4.40–7.75)
Happiness	92.21	4.87	(82.42–102.00)	38.26	3.39	(31.44–45.08)	3.78	0.63	(2.53–5.04)
Sadness	98.12	7.39	[83.25–112.98)	61.84	6.23	[49.31–74.37)	6.74	0.92	[4.89–8.59)

## Data Availability

The original data presented in this study is openly available at https://doi.org/10.7910/DVN/8SU8KN.
